# Case Report: Detailed Clinical Course and Management Plan for Status Epilepticus Pediatric Patient with Resected Choroid Plexus Papilloma: A Case Report and a Single Center Experience

**DOI:** 10.12688/f1000research.122349.1

**Published:** 2022-06-23

**Authors:** Osama Muthaffar, Anas Alyazidi, Fahad Alotibi

**Affiliations:** 1 Department of Pediatrics, King Abdulaziz University, Jeddah, Saudi Arabia; 2 Faculty of Medicine, King Abdulaziz University, Jeddah, Saudi Arabia

**Keywords:** Pediatrics, Female, Choroid plexus papilloma; Epilepsy; Seizures; Ventricle

## Abstract

Choroid plexus papilloma (CPP) is a benign but rare central nervous system (CNS) neoplasm of the choroid plexus. The onset of symptoms is usually in the first decade and may occur at birth (i.e., congenital). It accounts for 0.4–0.6% of all brain tumors. Usually seen in patients who are young children. The object of this clinical case to highlight early surgical intervention, intensive and multidisciplinary care, and pharmaceutical prescriptions can enhance the patient’s condition and quality of life. We herein report a rare presentation of CPP in a 6-year-old Sudanese female child with seizures. Who suffered from obstructive hydrocephalus with lateral ventricular choroid plexus papilloma. The patient underwent resection at the age of 6 months in our center’s neurosurgery department. Intensive and multidisciplinary follow-up managed to maintain positive outcome and better quality of life in a relatively benign neoplasm. In spite of a wide range of therapeutic options for the management of CPP described in the literature, studies demonstrated that patients with CPP alone and underwent a surgical procedure can live independently as adults and work full-time with uncommon recurrences.

## Introduction

Choroid plexus papilloma (CPP) is a rare benign neoplasm of the choroid plexus that accounts for 0.4–0.6% of all brain tumors. ^1^ It is a vascular neuroepithelial tumor The choroid plexus is a structure made from tufts of villi within the ventricular system that produces cerebrospinal fluid (CSF). ^2^ CPP most commonly presents in the lateral ventricles in the pediatric population. ^3^ Nonetheless, extra-ventricular sites have been described in the literature. ^4^ CPP can be detected in all age groups, however, there is a particular preponderance among infants and extremely young children with a median age of 3.5 years at diagnosis. ^5^ Compressive activity on the certain horns of the lateral ventricles could subsequently lead to developing seizures. ^6^ Moreover, current treatment of choroid plexus tumors (CPT) is based on little evidence ^7^ and the clinical management of CPT cases could be complicated if the presented patient is in the pediatric population and simultaneously experiencing epileptic seizures. On this note, we presented a detailed clinical management experience of a single patient with resected CPP in her early life and currently diagnosed with status epilepticus. To the extent of our knowledge, this is the first detailed clinical management report by a tertiary care center in Saudi Arabia.

## Case report

### Ethical approval

Ethical approval is not required at our institution to publish an anonymous case report. Written informed consent for publication of their clinical details was obtained from the patient.

Following the CARE Guidelines, a six-year-old Sudanese female child presented to the emergency department (ED) with a history of an uprolling eye for two minutes. The mother is a 45 year old housewife, and the father is a 55 year old. She has one sister and four brothers, and all are healthy. Negative history of parents’ consanguinity. The mother was following up during her pregnancy with no history of either passive or active smoking, no history of diabetes mellitus (DM) or hypertension, and no history of using any medications. Prenatal, natal, and post-natal care was given. The child is a product of full-term, spontaneous vaginal delivery (SVD) with a birth weight of 4.5 kg, discharged with the mother in good conditions. At five months, the mother noticed head enlargement and sought medical advice. She was diagnosed with obstructive hydrocephalus with lateral ventricular choroid plexus papilloma underwent resection at the age of six months in our center’s neurosurgery department. Postoperative computed tomography (CT) included frontal craniotomy and large extra-axial pneumocephalus with large frontal air-fluid levels causing a mass effect on the brain more dominant in the left hemisphere. A previously noted large cauliflower-like mass in the left ventricle has been resected with multiple small hyperdense masses that could represent residual. Hyperdense area anteriorly within the fluid on the left extra-axial space and another adjacent to the left parietal-temporal lobe most likely representing postoperative hemorrhage. No Acute intra-axial hemorrhage. No acute ischemic insult. Less dilatation of ventricular system as compared to the previous imaging. Periventricular hypodensity likely related to spontaneous fracture (SF) permeation was noted again. A small intraventricular hemorrhage is noted with air in both temporal horns. Cystic area in the posterior fossa communicating with the fourth ventricle with no mass effect consistent with mega cisterna magna. External ventricular drain (EVD) tip was noted in the anterior left lateral ventricle with a right frontal approach. Left frontoparietal-temporal subcutaneous swelling and air are associated with two fractures of the left parietal bone. The patient underwent her first magnetic resonance imaging (MRI) postoperatively. Multiple axial, sagittal, and coronal sequences of the brain with and without IV contrast were used. With no previous MRI examinations available for comparison, a comparison was made with the last examination, a CT study that revealed marked irregular supratentorial ventricular dilatation, which is increased. No MRI evidence of residual or recurrent neoplastic tissue. Diffuse pachymeningeal enhancement is most likely related to meningeal irritation and not due to leptomeningeal metastasis. Left parietal brain gliosis related to previous operative interference. It is still seen as bilateral subdural fluid; however, it is less than seen in the previous examination. No midline shift, no mass effect, no intraventricular hemorrhage and no other significant abnormality was identified. Moreover, at the age of four-year-old, she developed a seizure in the form of focal eye movement. She started on Keppra, requiring pediatric intensive care unit (PICU) admission once last year as a case of status epilepticus. Regarding her current ED visit, the patient was in her usual state of health until 9:30 AM, when she started to have abnormal uprolling eye movements while being fully awake during the episode with normal limb movement and communicating with her mother. The first episode aborted by itself. Furthermore, according to her legal guardian, the patient experienced multiple attacks of vomiting with food content, not projectile. Upon arrival in the ED, she developed another attack with desatting to 70% in room air. However, the second episode aborted after administering diazepam (20 mg/kg). The patient was vitally stable, drowsy, and communicating with her legal guardian. CT did not reveal significant or acute changes or other interval changes besides the previously resected brain tumor, which resulted in dilated lateral ventricles compared to her right ventricles. On physical examination, her pupils were equally reactive to light, she was hypotonic in the right upper limb with normal tone in other limbs, and had hyperreflexia and negative clonus and a negative Babinski sign (plantar reflex). The requested tests included complete blood count (CBC), urea and electrolytes (U&E), and bone function, where they were all within normal range. The case was discussed with a pediatric neurology consultant, and she started on Keppra 30 mg/kg/day divided twice a day (BID) with follow-up in the clinic. The patient was discharged once fully awake. A week later, the patient followed up on her seizure, where she only had four attacks in a previous couple of years in the form of a complex partial seizure. She remained on Keppra with the same dosage and was discharged. Ten days later, she presented to the ED complaining of a seizure lasting for ten minutes with eyes deviating to the left side, according to her legal guardian. The patient presented in post-ictal status in the ED with no significant systematic findings. She was vitally stable with a triage category three until she suddenly deteriorated to 80% in room air, respiratory rate (RR) 12 breaths per minute (BPM), heart rate (HR) 114 with on and off eye deviation to the right side. However, her pupils were three mm reactive to light. The patient, however, was shifted to the resuscitation room and connected to oxygen, and given intravenous (IV) diazepam of six mg (0.2 mg/kg). The patient was well hydrated, with shallow breathing with no added sounds or heart murmurs, and the abdomen was soft and lax with no tenderness or organomegaly. Ambu bagging was initiated, and PICU was contacted for further profound care. During the time, the patient’s pupils were bilaterally equal and reactive to light and maintaining oxygen saturation at 100%. Upon PICU team arrival, the patient developed generalized clonic status epilepticus, code announced, and then the patient was intubated for one day only. Phenytoin and Keppra were given in a loading dose, then started on midazolam infusion of 1 mg/kg/hr and fentanyl as sedation until she was extubated 24 hours later. The patient was stabilized and transferred to the PICU. She did not experience any epileptic seizures and was sleepy. On the next morning (7 AM), the patient entered a state of metabolic acidosis resulting in intervening with four plus 10/kg of Lasix (furosemide) in addition to vancomycin for two doses before discontinuing it. On the next day, the patient was transferred to the medical ward. On the fourth day of the recent admission, the patient’s vitals were as follows: body temperature (Temp.) 36.5, heart rate (HR) 86, oxygen saturation (SaO _2_) 99% at room air, and blood pressure (BP) 99/50. Body weight (BW) was 30 kg and height (Ht.) 138 cm. No distress or complaints, according to her legal guardian. Upon central nervous examination, she was Keppra 40 mg/kg/day BID, conscious and alert, right-sided weakness with hyperreflexia, normal tone and power on the left side, pupil bilaterally equal and reactive to light, and no meningeal signs. She was hemodynamically stable with no systemic manifestations. On her follow-up with the infectious disease (ID) department, she started to receive ceftriaxone-D3 and vancomycin. C-reactive protein (CRP) was initially three mg/L, then repeated where it was 17.4 mg/L. She was discharged with culture pending, and neurological stabilization was achieved. Nine months later, the patient became seven-year-old, and she returned to the neurological clinic after her legal guardian noticed a developmental delay in the child. The last seizure episode was two months earlier after she had another episode nine months earlier. During this visit, she was conscious of the normal motor exam. However, she was diagnosed with hydrocephalus and developmental delay. A plan to increase Keppra dosage to seven ml BID was made and titrated to eight ml if seizures are not controlled. She was discharged and returned six months later to conduct a developmental assessment as she became an eight-year-old. Her developmental history started with expressive language assessment: At an early age, around two-to four, she requested things by using the other hand with no eye contact and no joint attention. She started to talk at the age of four years with few words MAMA and BABA were unspecific, then she started to improve at the age of six years after enrolment in speech session in a specialized center, at that time till now she can say three words sentences mainly order with no eye contact and no joint attention, and she still cannot run conversions. She cannot answer only specific simple questions and only her mother when she repeats them many times. Receptive assessment: The patient only obeys simple commands. She can sometimes obey two steps for specific things that she likes (go to bring an orange, clean it, and cut it Into pieces). Sometimes she repeats other words (Echolalia). Socially, she initiates playing with younger children. She likes no specific toys, only she likes to run, scream, and produce unspecific voices, and sometimes she expresses playing by hitting the younger children. Routine and sensory: According to the mother, she used to flap with her hand at the age of one-two years which disappeared at the age of four years, and currently no specific repetitive pattern. She likes specific clothes, and when she does not find them, she cries. Other sensory fields, including visual, hearing, and taste, were unremarkable. Emotionally: She does not understand others’ emotions. When she sees others crying, she usually laughs. Cognitively: She does not understand the concept of danger. She usually runs in the street with no sense of fear of cars or being cautious. Sleep: She sleeps for roughly eight hours from 1:00 AM till 10:00 AM, with no interruption and no nap. Media: She likes mobile most of the time. She likes to watch and listen to music. School: She started rehabilitation and mainly physiotherapy for the motor aspect as early as two years. Then at the age of four, she started speech and behavior modification sessions in a specialized center. She quit the rehabilitation center for more than one year because the family had traveled to Sudan. On physical examination, her weight was 37 kg, height 138.6 cm, all in the 95th percentile, and head circumference 57 cm above the 95th percentile. Her vitals were Temp. 37, HR 85, RR 22, BP 90/53, SaO _2_ 100% at room air. Her laboratory work was as follow: white blood cell (WBC) count 8.02 k/μl (reference range: 4-11 k/μl), red blood cell (RBC) count 3.99 M/μl (reference range: 4.8-6.4 M/μl), hemoglobin (Hb) 10.6 g/dl (reference range: 13-17 g/dl), hematocrit (HCT) 31.8% (reference range: 41%-50%), mean cell volume (MCV) 79.7 fl (reference range: 76-92 fl), platelet (PLT) count 235 k/μl (reference range: 150-450 k/μl), prothrombin time (PT) 12.4 seconds (reference range: 11.7-15.3 seconds), activated partial thromboplastin time (APTT) 28.1 seconds (reference range: 28.9-38.1 seconds), and international normalized ratio (INR) 1.11 seconds (reference range: 0.89-1.18 seconds). On clinical observations, she looked well, with no dysmorphic features, no eye contact, and sat at the clinic’s beginning, calm, watching online videos. However, after 30 minutes, she started to clean her hand around five times, then after 45 minutes, she started to scream and produce nonspecific sounds. She does not like to draw or use any toys in the clinic. She is only interested in mobile phones, and when her sister took them from her, she cried and hit her sister. No systematic findings. However, her current presentation was consistent with Autism Spectrum Disorder (ASD). Her 8th and most recent CT scan redemonstrated a dilatation of the supratentorial ventricular system, with stable porencephalic cystic changes adjacent to the left lateral ventricle. Preserved gray-white matter differentiation. No extra/intra-axial hemorrhage or herniation. The posterior fossa structures are unremarkable. The visualized osseous and orbital structures are unremarkable. The paranasal sinuses and mastoid air cells are well aerated. In conclusion, her imaging work-up displayed a stable appearance, without acute findings or other interval changes. Her second MRI imaging, which used a non-enhanced sagittal T1W, axial T2W, FLAIR, DW, GE, SW, and coronal T2W images of the brain, were obtained with axial, sagittal, and coronal T1W after IV contrast administration. A comparison was made based on her previous MRI. Findings included ventricular enlargement and irregularity of the left lateral ventricle are stable in appearance. Cystic structure communicating with the left lateral ventricle is also unchanged. A small amount of periventricular increased signal in the left dorsal thalamus is unchanged. No new enhancing ventricular or parenchymal lesions. Near complete resolution of the left frontal subdural hematoma with a small residual right frontal subdural collection remaining, measuring a maximum of three mm on the axial plane. She was on keppra (levetiracetam) 100 mg/mL, oral solution 300 mt, phenytoin Na 250 mg/5 mL ampule, and carbamazepine 200 mg tablets. Her management plan included extensive speech and behavior modification sessions through enrollment into rehabilitation centers and encouraging the family to be part of behavior modifications and follow-up after six months.

## Discussion

We reported a case of a resected lateral ventricle CPP presenting with seizures, to the extent of our knowledge, it is the first case to be reported from Saudi Arabia. The brain tumor which accounts for 0.4–0.6% of all brain tumors. ^8^ Demonstrated to create a puzzling clinical course which requires multidisciplinary intervention. Having this condition in a pediatric patient demands caregivers to develop detailed management plans that takes into consideration physical and psychological aspects of the child for a better quality of life. The median age for diagnosing CPP is 3.5 years, ^5^ however, our patient was diagnosed very earlier. Although there was no indication of CPP on our patient’s MRI at the corrected age of 34 weeks’ gestation, because no contrast scan was performed, it is difficult to rule out the presence of a minor lesion at the time. As a result, the majority of tumor growth occurred within the five-month period between the two MRI tests, confirming that certain CPP grow rapidly throughout early childhood. Such observation is indirectly supported by the finding that in the literature prenatal detection of CPP is relatively infrequent compared to the presentation of a substantial CPP in the first year of life. ^9^ The motives behind certain CPP growing rapidly during childhood is still unknown. CPP can also grow because to tumor hemorrhage and malignant progression. Furthermore, CPP can develop to a big size regardless of its ventricular position, it appears logical to believe that tumor location and ventricular size may affect the rate of growth. ^9^^,^^10^ Moreover, MRI testing is an essential part when investigating hydrocephlus which was presented in our case. Intractable epilepsy etiologies must be closely monitored to maintain the patient’s health, literature findings suggests that intracranial hypotension could cause intractable epilepsy in pediatric patients with resected CPP. ^11^ Continuous treatment, dose adjustment according to the patient’s condition and repeated clinic visit proved to be a successful approach in advancing the patients’ outcome. Finally, the diagnosis of pediatric brain tumors has long been regarded as a serious threat to children and their families, due to poor prognosis in certain tumors. however, according to our experience, intensive and multidisciplinary follow-up for our patient managed to maintain positive outcome and better quality of life in a relatively benign neoplasm. According to studies with broader subjects, the short-term survival rates for CPP are optimistic after a surgical intervention alone, nonetheless, clinical results were disappointing. ^12^ It is wise to say that when the patient experiences a chronic condition such as epilepsy could complicate the management plan. Long-term survival and quality of life for CPP patients with epilepsy remains an area of research, however, studies demonstrated that patients with CPP alone and underwent a surgical procedure can live independently as adults and work full-time with uncommon recurrences. ^8^

## Conclusion

We presented one of the most detailed clinical reports of a CPP patient with epilepsy. It is important to highlight daily practices for managing this condition. We reported a positive outcome for a patient diagnosed very early in her life and today lives in her 10th years of age in her usual state of health. Our report confirms that early surgical intervention, intensive and multidisciplinary care, and pharmaceutical prescriptions can enhance the patient’s condition and quality of life. Repeated and justified use of MRI and CT showed to be of great interest in detecting other comorbidities. This case demonstrated great challenges in terms of follow-up, management, and caregiving. In the future, we recommend more inclusive guidelines that take into consideration epilepsy during the management of CPP.

## Consent

Written informed consent for publication of their clinical details and/or clinical images was obtained from the patient/parent/guardian/relative of the patient.

## Data availability

### Reporting guidelines

Figshare: CARE checklist from the case report entitled “detailed clinical course and management plan for status epilepticus pediatric patient with resected choroid plexus papilloma: a case report and a single center experience.” DOI: https://doi.org/10.6084/m9.figshare.20029580.v1. ^13^

Data are available under the terms of the Creative Commons Attribution 4.0 International license (CC-BY 4.0).
