# Supplementary material for: Case Report: Detailed Clinical Course and Management Plan for Status Epilepticus Pediatric Patient with Resected Choroid Plexus Papilloma: A Case Report and a Single Center Experience
Source: F1000Res. 2022 Jun 23;11:695. [Version 1] doi: 10.12688/f1000research.122349.1 (PMC10622857; doi:10.12688/f1000research.122349.1)
Supplement: Peer review history

# Reviewer report: Reviewer response for version 1

**DOI:** 10.5256/f1000research.134327.r201416

**Published:** 2023-11-02

**Authors:** Aaron Mohanty

**Affiliations:** 1 UTMB Health Neurosurgery, Galveston, Texas, USA

The authors have reported a case of left lateral ventricular choroid plexus papilloma (CPP) in a 6-year-old girl which was resected initially at 6 months of age and presented subsequently with multiple seizures at 4 years of age requiring anticonvulsants.  At follow-up, she was noted to have a developmental delay.

 As the authors mention, CPP is a childhood tumor but can be also seen in adults. It is one of the few tumors which is accompanied by increased CSF production with hydrocephalus. The large ventricles aid in the surgical approach with the dissection easier than an intraparenchymal tumor. The large ventricles also permit an endoscopic approach to the tumors and there are several reports where these tumors have been resected by a pure endoscopic approach. Asymptomatic subdural collections and intraventricular bleeds are common in the postoperative period and often a ventricular drain is required.  Persistent postoperative ventriculomegaly may require a CSF shunting procedure. Though total excision is possible, it is not uncommon to leave a small amount of tumor in close proximity to the periventricular vital venous structures. Children may develop seizures in the postoperative period which often can occur late. Close follow-up will be required to detect any recurrences. The overall morbidity is associated with the surgical approach in the young developing brain and associated blood loss, degree of hydrocephalus, residual tumor, and any chronic anticonvulsant usage.

 In recent years, some  choroid plexus papillomas are resected by a minimally invasive endoscopic burr hole approach which significantly reduces postoperative morbidity. The vascularity of the tumor often can be a concern ^1^.

 Recent genetic studies have reported an association of CPP with  Aicardi syndrome; von Hippel–Lindau syndrome; and infrequently with Li–Li-Fraumeni and rhabdoid predisposition syndrome.

Are enough details provided of any physical examination and diagnostic tests, treatment given and outcomes?

Yes

Is the case presented with sufficient detail to be useful for other practitioners?

Yes

Is sufficient discussion included of the importance of the findings and their relevance to future understanding of disease processes, diagnosis or treatment?

Yes

Is the background of the case’s history and progression described in sufficient detail?

Yes

Reviewer Expertise:

Pediatric Neurosurgery,  minmallly invasive endoscopci neurosurgery,

I confirm that I have read this submission and believe that I have an appropriate level of expertise to confirm that it is of an acceptable scientific standard.

## References

[1] : Third ventricular choroid plexus papilloma: a pure neuroendoscopic approach. *Neurosurg Focus Video* .2023;8(2) : 10.3171/2023.1.FOCVID22145 V8 10.3171/2023.1.FOCVID22145 37089747PMC10114913

# Reviewer report: Reviewer response for version 1

**DOI:** 10.5256/f1000research.134327.r141966

**Published:** 2022-06-27

**Authors:** Ayman Zaky Elsamanoudy

**Affiliations:** 1 Clinical Biochemistry, Faculty of Medicine, King Abdulaziz University, Jeddah, Saudi Arabia; 2 Medical Biochemistry and Molecular Biology Department, Faculty of Medicine, Mansoura University, Mansoura University, Egypt

The authors provided a full description of the case and its management. They provided a full explanation in the discussion section. Moreover, they provided a satisfactory conclusion and recommendation.

 These comments to be addressed:

1. Abstract: includes only the background of the disease. I recommend adding the aim of the study and the conclusion to give concise information about the context of the case report.
2. 2- They mentioned that(To the extent of our knowledge, this is the first detailed clinical management report by a tertiary care centre in Saudi Arabia.).They have to mention that the patient is not a Saudi but she is Sudanese but by chance, the management was in Saudi Arabia.

Are enough details provided of any physical examination and diagnostic tests, treatment given and outcomes?

Yes

Is the case presented with sufficient detail to be useful for other practitioners?

Yes

Is sufficient discussion included of the importance of the findings and their relevance to future understanding of disease processes, diagnosis or treatment?

Yes

Is the background of the case’s history and progression described in sufficient detail?

Yes

Reviewer Expertise:

Medical Biochemistry and Molecular biology, Molecular aspect of metabolic syndrome and its related genetic metabolic disorders.The genetic and molecular aspects of cancer.

I confirm that I have read this submission and believe that I have an appropriate level of expertise to confirm that it is of an acceptable scientific standard.
